# The role of vision for navigation in the crown-of-thorns seastar, *Acanthaster planci*

**DOI:** 10.1038/srep30834

**Published:** 2016-08-01

**Authors:** Robert Sigl, Sebastian Steibl, Christian Laforsch

**Affiliations:** 1 Department Animal Ecology I, University of Bayreuth and BayCEER, Universitaetsstr. 30, 95447 Bayreuth, Germany

## Abstract

Coral reefs all over the Indo-Pacific suffer from substantial damage caused by the crown-of-thorns seastar *Acanthaster planci*, a voracious predator that moves on and between reefs to seek out its coral prey. Chemoreception is thought to guide *A. planci*. As vision was recently introduced as another sense involved in seastar navigation, we investigated the potential role of vision for navigation in *A. planci.* We estimated the spatial resolution and visual field of the compound eye using histological sections and morphometric measurements. Field experiments in a semi-controlled environment revealed that vision in *A. planci* aids in finding reef structures at a distance of at least 5 m, whereas chemoreception seems to be effective only at very short distances. Hence, vision outweighs chemoreception at intermediate distances. *A. planci* might use vision to navigate between reef structures and to locate coral prey, therefore improving foraging efficiency, especially when multidirectional currents and omnipresent chemical cues on the reef hamper chemoreception.

The crown-of-thorns seastar, *Acanthaster planci*, is an abundant echinoderm (Asteroidea: Valvatida) in coral reef communities of the Indo-Pacific^1^. Unlike most seastars, *A. planci* mainly feeds on reef-building corals^2^. This coral diet, in combination with its frequent occurrence in high-density populations (outbreaks), render *A. planci* as one of the major threats for Indo-Pacific coral reefs^3^,^4^. Within outbreak populations, individuals of *A. planci* often align in feeding fronts that move continuously to previously unexploited areas of the infected coral reef 5,6. Especially in reefs with low coral cover and therefore an increased intraspecific competition for food, the foraging movement of single *A. planci* is accelerated to invade undamaged, coral-bearing areas^7^. To avoid areas that have already been preyed on, directional movement towards new food sources can be beneficial, minimizing energy consumption to optimize foraging efficiency^8^. However, moving on a directional path requires a reliable cue^9^. *A. planci* is thought to be guided primarily by chemical cues released by corals^10^,^11^. To detect these prey odours, *A. planci,* like all seastars, is equipped with chemoreceptors that are concentrated on sensory tube feet at the distal end of each arm^12^. Its chemoreceptors are thought to allow *A. planci* to detect and navigate towards its food source and to discriminate between preferred (e.g. *Montipora* spp.) and rejected (e.g. *Porites* spp.) coral prey species^13^,^14^,^15^. When chemical cues of coral prey are present in the water, seastars usually move upstream and follow the chemical gradient towards the food source^16^,^17^,^18^. However, in the absence of chemical cues, seastars are still capable of directing their movement upstream or downstream, i.e. rheotaxis^18^. Many seastar species thereby display either a cross-current movement to maximize their chances of prey encounter, or a downstream movement to minimize energy consumption^18^,^19^,^20^. It has been suggested that seastars use basic mechano-sensitive nerve endings in the cuticle to determine the current direction^21^,^22^. However, turbulent environments such as coral reefs constrain both rheotaxis and chemotaxis^16^. Multidirectional currents and intricate flows cause a complex and spatially unpredictable signal and unreliable cues. At the same time, they strongly reduce tracking distances, because directional transport of the chemical cues is diminished^23^,^24^.

Considering the spatially very restricted reliability of chemical cues^25^ and water currents among reefs, *A. planci* probably uses other senses such as vision. As in all seastars, each arm tip of *A. planci* bears one compound eye, or optic cushion, situated at the base of a modified tube foot and consisting of several ommatidia^26^. In addition to compound eyes, seastars possess a dermal light sense, thought to arise from photosensitive superficial nerves^27^. Garm and Nilsson^28^ recently showed that the seastar *Linckia laevigata* uses its eyes for basic navigation. It remains unknown if vision outweighs chemoreception as a reliable sense for navigation at certain spatial scales.

## Methods

### Spatial resolution and visual field of the compound eye

Adult *A. planci* were collected on the coral reefs of Moorea, French Polynesia. From each of the nine individuals collected, one arm tip was preserved with 8% formaldehyde in seawater. Compound eyes were dissected using a scalpel and fine scissors. Four eyes were embedded for semi-thin sectioning to analyse the acceptance angles of the single ommatidia. The other five eyes were used for the approximation of the visual field, as well as the identification of the total number of ommatidia per compound eye and the interommatidial angles. Eyes were decalcified in EDTA, dehydrated in a graded acetone series and embedded in epoxy resin so that semi-thin sections could be examined (Axio Scope.A1, Carl Zeiss AG, Ulm, Germany; Camera: AxioCam MRm, Basler AG, Ahrensburg, Germany). Embedding and sectioning procedures are fully described in online Supplementary Information. Three ommatidia of each of the four eyes were chosen randomly and the maximum width and depth was measured using ImageJ 1.49b^29^. The measurement of the minimal and maximal acceptance angles of the single ommatidia was conducted following the procedure of Garm and Nilsson^28^ using ImageJ 1.49b. The acceptance angles were used to approximate the minimal and maximal vertical and horizontal visual field by adding them to the angle between the optical axes of the two outermost ommatidia in each orientation. To measure this angle, the dissected eyes of one small (ca. 15 cm diameter) and four large (30–50 cm diameter) *A. planci* were placed under a microscope (Olympus BX63, Olympus Corp., Tokyo, Japan; camera: Olympus MX10) and photographed from a lateral view. The eyes were then bisected axially and the cross sectional planes were imaged. The interommatidial angles (i.e. the distance in degrees between the optical axes of neighbouring ommatidia of the eye) of 53 ommatidia along the horizontal midline and of 38 ommatidia along the vertical midline from the same eyes were measured using ImageJ 1.49b. Based on these angles, the average interommatidial angle was calculated using the equation^30^:


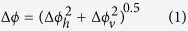


where Δ*ϕ*_*h*_ is the interommatidial angle along the horizontal midline of the compound eye and Δ*ϕ*_*v*_ is the interommatidial angle along the vertical midline. Top-view images of the whole compound eye were used to count the number of ommatidia. Ommatidia of one half of the bilaterally symmetric compound eye were counted using ImageJ 1.49b (Multi-Point Tool) and this number was doubled to obtain an estimate for the total number of ommatidia.

## Field Experiments. 

### Experimental animals and area

*A. planci* were collected at different locations around the island of Moorea, French Polynesia and transported to the R.B. Gump Station where they were kept in large plastic bins supplied with running seawater from the ocean without food for 2–4 weeks. Field experiments took place in November and December 2013 at Temae beach (17°29′52.43″S; 149°45′28.61″W) on Moorea. The experimental area was located in a large lagoon with sandy ground and few interspersed living coral heads at a water depth of about 2.5 m. It was situated approximately 50 m from the shore and 370 m from the surrounding atoll reef crest.

## Eye amputation

Individuals were anaesthetized using 3.5% magnesium-chloride-hexahydrate (MgCl_2_ × 6H_2_O) in seawater to prevent movement. The terminal ossicle together with the harboured terminal tube foot containing the optic cushion at its basis was carefully cut off from each arm tip using a pair of fine scissors. After surgery, individuals were placed in the aforementioned bins and allowed to recover until the next day.

## Preliminary experiment on movement direction and impact of eye amputation

To test for the general movement direction of *A. planci* in the experimental area a preliminary experiment was conducted close to the area of the main experiment to exclude any influence of the eye amputation on behaviour. An area with the closest potentially perceivable coral at a distance of approximately 50 m was chosen. There, an influence of chemical cues released by food on movement behaviour was unlikely. In each trial one blinded and one non-blinded *A. planci* (N = 5) were placed in a small shelter artificially built with dead coral rock collected within a radius of 150 m. The animals were allowed to move freely for one hour on sand, then tracked using GPS (GPSMap 60 CSx; Garmin Ltd., USA). The displacement and direction of movement were analysed using Arc-Map (ArcGIS 10.2.1, ESRI, Redlands, USA). The current direction was determined to the nearest 5° using a compass and a plastic flag.

## Experiment on visual navigation

**Figure 1:**
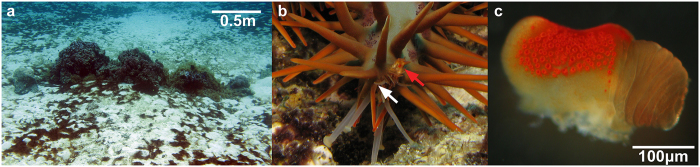
Experimental area and eyes of *A. planci*. (**a**) Reef structure used in the main experiment. (**b**) Arm tip of a moving *A. planci*; red arrow indicates the compound eye and the white arrow the extended sensory tube feet. (**c**) Compound eye of a small *A. planci*.

During the first two weeks of December 2013, a reef structure with live coral (mainly *Montipora* spp., some *Porites* spp.) perpendicular to the direction of the current was selected. The structure was about 2.5 m wide and consisted of a row of five differently sized heads up to 0.5 m in height above ground (Fig. 1a). Water currents flowed constantly in one direction more or less parallel to the shore, independent of the tides. A unidirectional current was needed to carry consistent chemical cues towards the experimental animals. Small pieces of coral rubble were used to mark starting points 1.25 m, 2.5 m, 5 m and 10 m downstream of the reef structure. For each trial one blinded and one non-blinded adult *A. planci* (25–50 cm diameter) were placed next to each other at the starting point and allowed to move freely while being observed from distance (N = 10, except for 5 m distance: N = 11). Trials were terminated as soon as the animals had moved more than twice their initial distance to the reef structure, after half an hour of observation or if the seastars reached the reef structure and started to climb it, at which point the position relative to the starting point of each *A. planci* was determined using a compass. After that, the seastars were removed and the next trial was conducted. The direction of the water current was determined to the nearest 5° using a compass and a plastic flag.

## Statistical analyses

Movement data from the preliminary experiment were analysed in SPSS Statistics 22.0.0.0 (IBM Corp., Chicago, USA) using a one-way ANOVA. Prior to analysis, data were checked for homogeneity of variances and normality. Directionality of movements was analysed in PAST 3.10^31^ using Rao’s Spacing test. This test was performed because the data violated the von Mises distribution assumption and it can handle the diametrically bimodal circular distributions present here^32^. Since all individuals walking inside an angular range pointing towards the reef structure had the opportunity to reach it, the directional data of the main experiment were grouped into sectors. The size of these sectors was chosen in a way that one sector contained the whole reef structure in its angle and its mean compass direction pointed towards 70°. This bearing represented the compass direction from the starting point to the middle of the reef structure. The size of the sector was adjusted depending on the distance to the reef structure so that one sector always fully contained the reef structure. Therefore, data from 1.25 m distance were grouped into three sectors with 120° angle, 2.5 m data into five sectors with 72°, 5 m data into nine sectors with 40° and 10 m data into 18 sectors with 20°. The mean compass directions of the respective sectors were used to analyse directionality of movement using Rao’s spacing test. Additionally, the frequencies of blinded and non-blinded individuals that reached the reef structure were compared using Fisher’s exact test in SPSS. If not stated otherwise all means are given with standard deviation (mean ± s.d.).

## Results

### Spatial resolution and visual field of the compound eye

**Figure 2:**
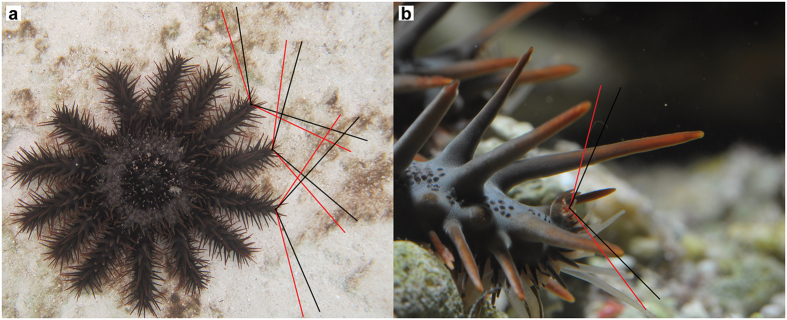
Visual field of *A. planci*. (**a**) Horizontal visual field. (**b**) Vertical visual field. Projected red lines represent the maximum visual field, black lines the minimum visual field.

The optic cushion of *A. planci* is conspicuously saddle-shaped. Semi-thin sections showed that ommatidia on the upper half are directed downwards to the substratum, whereas ommatidia on the lower half are directed upwards into the water column. Laterally situated ommatidia are orientated to their respective sides (Fig. 1c). The number of ommatidia of one compound eye of *A. planci* is variable and presumably dependent on the size of the individual. The large individuals (30–50 cm diameter) had eyes made up of 192–268 ommatidia, the small individual’s (approximately 15 cm diameter) eye consisted of only 130 ommatidia. The average distance of the optical axes of neighbouring ommatidia is 11.2 ± 5.9°. This interommatidial angle, however, varies from very dense areas of the compound eye by up to 3.2° to rather dispersed areas by up to 29.9°. The ommatidia themselves are 53 ± 12 μm long and 16 ± 3 μm wide. They have a minimum acceptance angle of 17 ± 4° and a maximum acceptance angle of 35 ± 9°. Based on the minimum and maximum acceptance angles of the single ommatidia, the determination of the visual field of *A. planci* revealed that the entire compound eye covers a minimum angle of 115 ± 15° and maximum of 132 ± 17° horizontally (Fig. 2a). Vertically, the compound eye surveys a minimum of 112 ± 33° and a maximum of 129 ± 24° (Fig. 2b). Due to the unusual saddle-shaped morphology of the optic cushion, the acceptance angles of the single ommatidia and therefore the whole visual field of a single compound eye are highly overlapping, especially in the area very close to the optic cushion. Furthermore, the projection of the horizontal visual field of an *A. planci* specimen revealed that the visual fields of the compound eyes obtain a 360° view and already overlap in an area very close to the animal (Fig. 2a).

### Field experiments

#### Preliminary experiment on movement direction and impact of eye amputation

Mean displacement during one hour on sand was 14.1 m ± 3 m for non-blinded and 15.0 m ± 2.6 m (both mean ± s.e.m.) for blinded individuals. Displacement of non-blinded and blinded individuals did not differ significantly (one-way ANOVA F_1,8_ = 0.052; p > 0.05). *A. planci* individuals showed significant directional movement (Rao’s Spacing test, U = 206.9; p < 0.01) to a mean direction of 242.2°, following the direction of the prevailing current (240°–270°).

#### Experiment on visual navigation

**Figure 3:**
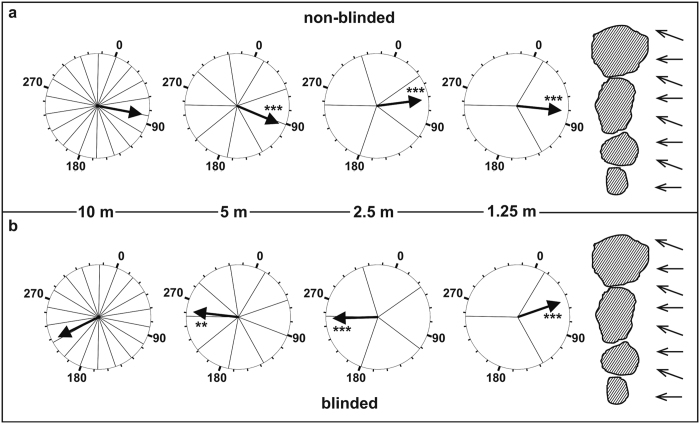
Mean direction of movement of non-blinded and blinded *A. planci* downstream of a reef structure. Mean compass directions of movement of *A. planci* released at different distances downstream a reef structure. (**a**) Non-blinded *A. planci*. (**b**) Blinded *A. planci*. Circles show compass directions in degrees and are orientated geographically. Arrows inside the circles point towards the mean direction of movement. Asterisks indicate the significance level of a preferred direction (**0.01; ***0.001). Hatched structures symbolize reef structure. Arrowheads to the right indicate the direction of the current.

The mean direction of movement in non-blinded animals was always towards the reef structure, i. e. upstream (mean direction of the water current: 250°–270°). In contrast, the mean direction of movement in blinded animals was always downstream at distances ≥2.5 m. Only at a distance of 1.25 m blinded *A. planci* moved towards the reef structure, i.e. upstream. Preferences for a specific direction in both treatments were significant at all distances besides at 10 m (Fig. 3, Table 1). One individual was excluded from the analysis (treatment: blinded, 2.5 m distance) as it was attacked by a pufferfish during the experiment, which may have biased its direction of movement.

From a distance of 2.5 m and 5 m, non-blinded *A. planci* found the reef structure significantly more often than blinded *A. planci* (Fisher’s exact test; p = 0.002 and p = 0.045, respectively). At a distance of 1.25 m more non-blinded individuals found the reef structure, but the number of successful navigations did not differ significantly from that of blinded individuals (Fisher’s exact test; p = 0.070). At 10 m distance, eyesight did not enable *A. planci* to find the reef structure significantly more often (Fisher’s exact test; p = 0.105). However, 30% of non-blinded individuals still found the reef structure. When the data over all distances are pooled, the ability to see strongly influences the ability to find reef structures (Fisher’s exact test; p < 0.001).

## Discussion

Although visual systems have been described in many echinoderms^33^,^34^,^35^, their ecological relevance often remained unclear. Recently, Garm and Nilsson^28^ provided first evidence for a general use of vision in seastars. In the present study, we present further evidence for the use of vision in behaviour of the predatory seastar species *A. planci*. Although its eyes possess relatively low spatial resolution, our results suggest that they play a significant role in orientation. Furthermore, our results allow a reassessment of the role of vision compared to chemoreception.

Compared to the eyes of other seastar species such as *Nepanthia belcheri*^26^ or *L. laevigata*^28^ the visual field of a single compound eye is about 50° smaller in *A. planci.* However, *A. planci* might still be able to maintain the same 360° view, as it can compensate for the smaller horizontal visual field of a single optic cushion by its increased number of arms (Fig. 2a). Vertically, the visual field of *A. planci* covers almost the complete water column, but leaves a blind spot directly above the central disk of the animal (Fig. 2b). Nevertheless, *A. planci* is still easily capable of surveying the water column directly above it by slightly bending its arm tip upwards, a behaviour commonly observed in moving seastars^12^. Hence, *A. planci* can possibly capture the whole area around and vertically above it simultaneously.

The relatively large interommatidial and acceptance angles of the compound eyes of *A. planci* suggest that they possess an overall low spatial resolution, as both angles are inversely proportional to the spatial resolution^36^. However, the ommatidia are not distributed equally over the surface of the eye. The central area of the compound eye in particular has nearly tenfold smaller interommatidial angles, suggesting that a higher spatial resolution can be achieved within this part of the eye. A comparable concentration of ommatidia can be found in insect eyes, where ‘acute zones’ give some parts of the eye an increased spatial resolution^30^. The saddle-shaped morphology of the optic cushion of *A. planci* furthermore causes highly overlapping interommatidial angles. This feature might reduce spatial resolution on the one hand, but could allow for a greater sensitivity to light on the other^37^. Such improved low-light vision might be beneficial for *A. planci*, which is normally nocturnal when occurring in low-density populations^38^,^39^.

As morphometric measurements can only give an estimate of what indeed can be resolved by the eyes, field experiments were required to support the measurements. In the preliminary field experiment, both blinded and non-blinded individuals moved similar distances, suggesting that the amputation procedure had no effect on movement behaviour. This is in accord with other studies showing no effect of eye amputation on the activity of seastars^28^,^40^. Additionally, the preliminary experiment revealed that the general movement direction of *A. planci* in the experimental area in the presumed absence of chemical cues from corals was downstream. Nickell and Moore^20^ described comparable downstream movement of the seastar *Asterias rubens* in the absence of chemical cues.

At a distance of 1.25 m, both non-blinded and blinded individuals moved upstream towards the coral bearing reef structure. This shows that the ability to see was not essential for the detection of the reef structure and confirms previous studies that found chemical cues to be used for navigating at short distances (reviewed by Sloan^25^). The source of these cues most likely were *Montipora* spp. present on the reef structure, which are a preferred diet of *A. planci* and have already been proven to induce a directional movement at short distances^13^,^41^. The importance of vision in finding the reef structure increased dramatically with distance. At distances of ≥2.5 m blinded *A. planci* displayed the same downstream movement as observed during the preliminary experiment, indicating that this already marks the spatial limit for chemoreception. In contrast, most non-blinded *A. planci* were able to find the reef structure when released at 2.5 m and 5 m. Yet, at a distance of 10 m only 30% of the non-blinded *A. planci* navigated towards the reef structure, indicating that this is close to the spatial limit of vision-mediated navigation. These results exceed our estimations based on the morphometric measurements, which predicted that *A. planci* could resolve an object of 1 m in size from 5 m distance. In comparison, *L. laevigata* possesses a much lower visual performance as it failed to detect a 3 m high reef structure from only 2 m distance^28^. *L. laevigata* is a rather slow-moving, microphagous grazer. As its food is highly abundant, it is not obliged to cover great distances and might use vision to ensure that it stays in its reef habitat while moving^28^. In contrast, *A. planci* is a relatively fast-moving predatory seastar that migrates between reef structures to reach unexploited feeding grounds^11^. Our results suggest that chemoreception is insufficient in these spatial scales. We propose that *A. planci* primarily uses its improved vision to find such reef structures.

Although vision is limited by visibility, it has the great advantage of being independent of water currents and distinct chemical gradients. Hence, as long as a reliable visual cue is present, vision could be used by *A. planci* for migrating between reefs, but also for navigating on the reef, therefore increasing overall foraging efficiency. Good vision could be beneficial for a predatory and mobile seastar like *A. planci,* but might play a minor role in grazing seastar species, suggesting an unequal relevance of vision in the biology of these animals.

## Additional Information

**How to cite this article**: Sigl, R. *et al*. The role of vision for navigation in the crown-of-thorns seastar, *Acanthaster planci. Sci. Rep.* **6**, 30834; doi: 10.1038/srep30834 (2016).

## Supplementary Material

Supplementary Information

## Figures and Tables

**Table 1:** Preferred direction of movement of blinded and non-blinded *A. planci* and percentage of successful navigation.

Distance [m]	Treatment	N	Circular mean	Rao’s U	P-value	% Successful
1.25	blinded	10	51°	252	**0.001**	50
	non-blinded	10	76°	288	**0.001**	90
2.5	blinded	9	248°	240	**0.001**	0
	non-blinded	10	63°	252	**0.001**	70
5	blinded	11	256°	196.4	**0.005**	0
	non-blinded	11	93°	229.1	**<0.001**	64
10	blinded	10	227°	172	0.050	0
	non-blinded	10	81°	140	0.355	30

Significant p-values of Rao’s spacing test are highlighted in bold.
