# Supplementary material for: The role of vision for navigation in the crown-of-thorns seastar, Acanthaster planci
Source: Sci Rep. 2016 Aug 1;6:30834. doi: 10.1038/srep30834 (PMC4967868; doi:10.1038/srep30834)
Supplement: Supplementary Information [file srep30834-s1.pdf]

Supplementary Information for:

The role of vision for navigation in the crown-of-thorns seastar, *Acanthaster planci*

Authors:

Robert Sigl; Sebastian Steibl; Christian Laforsch

Supplementary Methods

Preparation, embedding and sectioning procedure for the investigation of eye morphology

Decalcification of compound eyes was performed using 10 g EDTA, 1.7 g citric acid and 500 ml distilled water under 37° C in an overnight step. The eyes were then rinsed with 0.2 M phosphate buffer (230 ml 0.2 M NaH_2PO_4 x H_2O, 770 ml 0.2 M Na_2HPO_4, 1000 ml distilled water; 3x 30 min) at room temperature and afterwards dehydrated in a series of acetone (50%, 70%, 90%, 96%, 99.5%, 2x 10 min for each concentration, and 100%, 3x 20 min). Finally they were embedded in an epoxy resin using the following procedure: (I) 24 ml stock solution A (62 ml glycidether100, 100 ml DBA) and 18 ml stock solution B (100 ml glyicdether100, 89 ml MNA) were produced. (II) Stock solutions were mixed in a 4:3 ratio. (III) 0.76 g 1.5% DMP30 – epoxy accelerator was added and mixed at room temperature in an overnight step. (IV) Epoxy resins with the embedded eyes were polymerized at 60°C for 48 h. Subsequently, the resin blocks were trimmed (EM Trim, Leica AG, Solms, Germany). Serial semi-thin sectioning was performed (2 µm; UltraCut Microtome 701701 Ultramicrotome, Reichert-Jung, Vienna, Austria) using glass knifes (Ultramicrotome Knifeglas, 406 mm x 25.4 mm x 6 mm, Agar Scientific, Essex, UK) and sections were transferred to object slides. The samples were warmed for 2 min at 70°C and subsequently stained with Richardson-Blue [1] for 2 min at 70°C. Next, the slides were rinsed with distilled water and dried at 70°C for five minutes.

Supplementary References

1. Richardson, K. C., Jarett, L. & Finke, E. H. Embedding in epoxy resins for ultrathin sectioning in electron microscopy. *Biotech. Histochem.* **35,** 313–323 (1960).
